# A Phenomenological Study of Nurses’ Experience in Caring for COVID-19 Patients

**DOI:** 10.3390/ijerph19052924

**Published:** 2022-03-02

**Authors:** Hye-Young Jang, Jeong-Eun Yang, Yong-Soon Shin

**Affiliations:** 1 School of Nursing, Research Institute of Nursing Science, Hanyang University, Seoul 04763, Korea; white0108@hanyang.ac.kr; 2 Department of Nursing, Jesus University, Jeonju-si 54989, Korea; elfyang@jesus.ac.kr

**Keywords:** nursing, infectious diseases, caregiving, SARS-CoV-2, qualitative research

## Abstract

This study aimed to understand and describe the experiences of nurses who cared for patients with COVID-19. A descriptive phenomenological approach was used to collect data from individual in-depth interviews with 14 nurses, from 20 October 2020 to 15 January 2021. Data were analyzed using the phenomenological method of Colaizzi. Five theme clusters emerged from the analysis: (1) nurses struggling under the weight of dealing with infectious disease, (2) challenges added to difficult caring, (3) double suffering from patient care, (4) support for caring, and (5) expectations for post-COVID-19 life. The findings of this study are useful primary data for developing appropriate measures for health professionals’ wellbeing during outbreaks of infectious diseases. Specifically, as nurses in this study struggled with mental as well as physical difficulties, it is suggested that future studies develop and apply mental health recovery programs for them. To be prepared for future infectious diseases and contribute to patient care, policymakers should improve the work environment, through various means, such as nurses’ practice environment management and incentives.

## 1. Introduction

As the novel coronavirus disease (COVID-19) spreads worldwide and becomes more serious, the World Health Organization (WHO) has declared it a global epidemic. In Korea, the first case of COVID-19 was confirmed on 20 January 2020; as of 29 June 2021, the total number of patients was 156,167, of which 6882 were quarantined and treated, with a fatality rate of 1.29% [1].

COVID-19 is caused by a novel coronavirus—severe acute respiratory syndrome coronavirus-2 (SARS-CoV-2)—and manifests in clinical symptoms, such as cough (74.9%), fever (68.0%) and dyspnea (60.9%) among hospitalized patients [2]. In the case of SARS-CoV-2, it has been reported that if patients are isolated within 5 days of the onset of clinical symptoms, secondary infections occur less frequently; transmission can be effectively blocked by isolating immediately after the onset of symptoms [3]. However, hospitalizations in negative pressure isolation rooms, to block airborne infections, create a more isolated environment than the general intensive care unit environment; mandate medical personnel to wear unfamiliar and uncomfortable protective equipment; prohibit family visits and outside contact. Isolation affects patients as well, as it has been reported that many patients were insufficiently informed about the isolation environment and period, and this uncertainty caused them to experience depression [4]. These circumstances increase the importance of caring for patients in isolation.

Caring is an important concept within the field of nursing, as it affects the health of the patient as a whole [5]. In particular, in the early stages of outbreaks of new infectious diseases, all aspects, such as the pathology, transmission route, and effective treatment of the disease are uncertain [6]. Even the effectiveness of protective equipment is uncertain. It has been found that healthcare providers’ anxiety and fear in such conditions affects their ability to care for patients [7,8]. In the context of the COVID-19 pandemic, many scholars predict that the time before and after the pandemic will be very different and are asking if we are ready for post- or the ‘with COVID-19 era’ [9,10,11]. Even in nursing, this change is difficult to ignore, and nursing professionals and researchers should answer whether we are preparing the ‘with COVID-19 era’. In order to identify the reality of nursing in the ‘with COVID-19 era’, it is necessary to understand what nursing and caring experiences were like for nurses who have been care professionals during the unprecedented COVID-19 pandemic. During the pandemic, nurses played a positive role in the rapid reorganization of the nursing system, improvement of team communication, coordination materials for emergency and continuous care, improvement of efficiency of nursing performance as a front-line caregiver, and caring for other nurses [12]. However, nurses are starting to experience burnout, having been unaware that the pandemic would soon change health professions universally [13]. For this, it is necessary to examine the experiences of nurses who have been, and are, caring for quarantined patients.

Studies on the nursing experience of patients with COVID-19 are underway in countries in various trajectories of the COVID-19 pandemic, such as Spain [14], Italy [15], Canada [16], the United States [17], and China [18], and these previous studies are focused on the lived nursing experience itself or the ethical aspect. Experiences of nursing care reported so far are summarized as providing nursing care [14,15,16], psychosocial and emotional aspects [14,15,18,19], resource management [14,16], struggling on the frontline [19,20], personal growth [18,19] and adapting to changes [18,20].

In the context of the COVID-19 pandemic, the Korean government responded using the K-Quarantine, also known as 3T–Test (diagnosis/confirmation), Trace (epidemiological survey/trace) and Treat (isolation/treatment) [21]. In particular, since February 2020, COVID-19 hospitals have been designated and operated for safe isolation beds for hospitalization of COVID-19 patients [22]. As patients diagnosed with COVID-19 are transferred to a designated hospital, operating a medical system that receives intensive treatment and care, the nurses at the hospitals are facing a high level of depression, anxiety, and stress [23,24]. 

However, the nursing experience of Korean nurses is only a small part of the research done in the early stage of the pandemic, and that knowledge is not enough to understand the essence of nursing in the special nursing environment of COVID-19. Therefore, this study was conducted to understand the lived nursing experience of the nurses at COVID-19-designated hospitals during the third wave [25] of the COVID-19 pandemic in Korea. The nursing experience of Korean COVID-19-dedicated hospital nurses could provide a unique opportunity to develop long-term sustainable response strategies under a long-lasting pandemic.

Phenomenological research focuses on vivid experiences, perceived or interpreted by participants, and aims to view and describe the world of their consciousness as a real world. In addition, exploring the experiences of others can discover insights that were previously unavailable, so it is considered a useful method for the purpose of this study. Particularly, Colaizzi’s [26] method focuses on deriving a collection of common attributes and themes from multiple responses, rather than individual attributes. This method will facilitate an in-depth understanding of how nurses experienced caregiving for patients with COVID-19, and further contribute to the literature, regarding high-quality nursing care for quarantined patients. Therefore, the purpose of this study is to investigate the meaning and essence of nurses’ experiences of caring for COVID-19 patients, using a phenomenological research method. 

## 2. Materials and Methods 

### 2.1. Study Design

The philosophical framework and study design of this study were guided by phenomenology. The philosophical aim of phenomenology is to provide an understanding of the participant’s lived experiences [27]. In order to reveal the true essence of the ‘living experience’, it is first necessary to minimize the preconceived ideas that researchers may have about the research phenomenon (bracketing). Through such a phenomenological attitude, the participant’s experience can be explored as it is [28]. From a phenomenological point of view, objectivity is obtained by being faithful to the phenomenon, and it can be secured by paying attention to the phenomenon itself rather than explaining what it is. As such, phenomenology seeks to reveal meaning and essences in the participant’s experiences of the participant to facilitate understanding [28]. 

This study is an inductive study, applying the phenomenological research method of Colaizzi [26], in order to gain an in-depth understanding of the essence of nurses’ experience in caring for COVID-19 patients, and it followed the guideline for qualitative research, established by the Consolidated Criteria for Reporting Qualitative Research [29]. The question of this study is, “What is the meaning and essence of the care experience of nurses who directly cared for COVID-19 patients?”

### 2.2. Participants and Settings

Participants were nurses working at a COVID-19 Infectious Disease Hospital in Seoul and Gyeonggi Province. The COVID-19 Infectious Disease Hospital was established and is operated by the Ministry of Health and Welfare, one of the central government ministries of South Korea, to respond to infectious diseases during the COVID-19 pandemic. It is dedicated to managing infected patients. 

The inclusion criteria were as follows: nurses who had directly cared for confirmed or suspected COVID-19 patients in an isolation ward for at least 1 month; could communicate well and comprehend the purpose of this study; had voluntarily consented to participate. Nurses who had cared for COVID-19 patients for less than 1 month, had not participated in direct care, or had not been released from isolation, were excluded. Fourteen nurses participated in in-depth interviews individually (Table 1). 

### 2.3. Data Collection

Data were collected through in-depth interviews from 20 October 2020 to 15 January 2021 using purposive sampling (n = 12) and snowball sampling (n = 2). The sample size was determined by data saturation [30]. Data saturation was considered achieved when no new themes were revealed in the interviews of participants. Data saturation was determined by two researchers after the fourteenth case interview. Interviews were conducted either online or face-to-face by one well-trained researcher, depending on participants’ convenience. During face-to-face interviews, we created a comfortable atmosphere by beginning with everyday conversations. Interviews began with an open-ended question: “Tell me about your experience of caring for patients with COVID-19”, so that participants could elaborately and spontaneously describe their experiences. The interviews lasted about 60–120 min, and data collection and analysis were conducted simultaneously. 

### 2.4. Data Analysis

The interview content was transcribed verbatim within 24 h of each interview by the researcher. Transcripts of each participant’s interview and the memos were used to analyze data. Two researchers with doctoral degrees independently analyzed and discussed findings. 

Data analysis was guided by Colaizzi’s seven-step descriptive phenomenological method [26]: (1) researchers read all accounts multiple times to understand the overall flow of participants’ experiences in caring for COVID-19 patients; (2) we extracted significant statements from each description, focusing on meaningful statements related to participants’ caring experiences; (3) we formulated meanings from those significant statements, trying to discover the latent meaning in the context; (4) we organized those formulated meanings into themes and theme clusters; (5) the phenomenon under study was exhaustively described by integrating all the research results; (6) we identified the fundamental structure of the phenomenon; (7) finally, we validated this study by receiving feedback from two participants. 

In the entire process of data analysis, we tried to keep a distance from the researcher’s thoughts and feelings, and point of view about the phenomenon, as well as the content of the data, while being conscious of Husserl’s ‘bracketing’ [28]. In this way, we tried to avoid data distortion, reduction, and exaggeration by the researcher, and we tried to confirm and understand the perspective, attitude, and feeling of the participant as much as possible in the participant’s statement.

### 2.5. Rigor

To ensure trustworthiness of this study, the four criteria established by Lincoln and Guba [31] were used. For enhancing truth-value, we tried to obtain a rich set of data by selecting participants who would like to express the research phenomenon well and making it as comfortable as possible for the participants to state their experiences. We showed the study results to two participants to verify whether the derived results reflected the participants’ experiences. 

To ensure applicability, we provided the general characteristics of participants and tried to provide a thick description of the research phenomenon. 

To establish consistency, Colaizzi’s analysis method was adhered to, and the detailed research process and original data for each theme were presented to enhance the reader’s understanding of the research results. The researcher conducted the research while taking a neutral attitude throughout the research process, excluding bias, prejudices, assumptions (bracketing), so that the participant’s experience distortion by the researcher was minimized. In other words, in order to establish neutrality, which means freedom from prejudice about research results, at the beginning of the study, the researcher explicated any assumptions that could influence data collection and analysis [32] (ex. participants will mostly have negative emotions while caring for patients without any preparation. Participants will be withdrawn from the social perspective because they are taking care of infected patients.) The other researcher reviewed data analysis to ensure that the researcher’s assumptions did not influence data interpretation. 

### 2.6. Ethical Considerations

This study was approved by the Institutional Review Board of the researcher’s affiliated institution (HYUIRB-202009-009). Participants were informed about the purpose of the study, reporting of study results, and interview recordings. We obtained written informed consents from all participants before data collection. In addition, it was explained that even after consenting, participants could withdraw from the study at any time without any harm if they wished. All participants were provided with a small reward as appreciation for their participation in the study.

## 3. Results

The essential structure of the phenomenon was identified as ‘Going beyond the double suffering tunnel of taking charge of infected patients into the future’. The essence of the phenomenon is presented as five theme clusters, and twelve themes emerged from analyzing nurses’ experiences with caring for COVID-19 patients: (1) nurses struggling under the weight of dealing with infectious disease, (2) challenges added to difficult caring, (3) double suffering from patient care, (4) support for caring, and (5) expectations for post-COVID-19 life (Table 2).

### 3.1. Nurses Struggling under the Weight of Dealing with Infectious Disease

Participants felt fear and anxiety while caring for COVID-19 patients, as they have remained unaware of any definitive treatments. Consumed by thoughts of contracting the disease, they reported feeling unable to remain calm and dutifully serve their patients. In particular, it was shocking, as well as saddening, for them to be unable to provide respectful end of life care toward patients who could not recover.

#### 3.1.1. Anxiety and Fear Accompanying Patient Care 

The anxiety and fear at the heart of the thought that they could also be infected became an invisible chain, binding the participants. According to them, nursing without being guaranteed safety was challenging. When facing the reality of nursing while fearing patients’ diseases, it felt unfamiliar for participants to worry about their own and their patients’ safety simultaneously, rather than completely immersing themselves in patients’ recovery. They were uncertain of whether their feelings were normal; although they tried their best to provide quality care, they found it challenging to do so while dealing with their persistent anxiety.

*To be honest, that was the hardest for me. Since we were constantly exposed to the risk of infection, it was hard to care for patients due to anxiety rather than due to physical challenges while caring for the patient.*

(Participant J)

#### 3.1.2. Dignity Ignored Due to the Fear of Infectious Diseases

Having to watch patients struggling alone and in isolation, without the support and comfort of their family members during their final moments, made participants feel extremely sorry and heartbroken. The most distressing aspect of caring for patients on their deathbed was that patients and nurses were faced with the reality that patients’ families would not be allowed to be with them during their moment of dying; the fact that they would pass away without receiving appropriate treatment was secondary. “Patients who died during the COVID-19 period were the most pitiful” does not just indicate the limitations of medical treatment. It highlights dignity, which is be protected even in the worst circumstances, but was disregarded due to the fear of contracting infectious diseases. Participants experienced unimaginable shock and ethical anguish as they witnessed patients being taken to crematoriums without being seen by their family members, with their bodies in bags without having their clothing changed. As these uncontrollable experiences kept repeating, participants made a paradoxical resolve to prevent patients from dying.

*Patients who die while I work in the ward usually have their families come to see them and hold their hands. However, for those who die of COVID-19, families come and check their patients on the monitor. I think that’s the most heartbreaking and sad thing.*

(Participant L)

*The post-death process was really shocking. I feel like it didn’t treat people like human beings. Thus, that hurt me the most. I think that’s hard while working in the ward. When patients die, I know how they will be treated. I am so sorry, and my heart hurts. That’s why I really want to discharge them. Seriously, I think I’m getting desperate for this kind of feeling.*

(Participant B)

### 3.2. Challenges Added to Difficult Caring

Participants struggled every day, and factors that made their lives more challenging are as follows: the personal protective equipment (PPE) that had to be worn for patient care, working in chaotic conditions without clear instructions, and being overburdened with tasks.

#### 3.2.1. The Burden of Triple Distress for Everyone’s Safety; Wearing PPE

Participants had to endure a significant amount of pain and discomfort for safety purposes, especially while nursing patients in PPE. Less than 10 min after wearing them, the inside of the protective clothing would become warm and fill with sweat, and the eye goggles would become foggy. In these situations, participants experienced difficulties in certain activities, such as communicating with patients, securing intravenous (IV) lines, or drawing blood. Occasionally, they had to wear gloves that did not fit well due to a lack of proper supplies, making their practice more difficult.

*I think the hardest thing was to wear Level D and go inside. At first, I did the intubation wearing protective clothing. At that time, my body became sluggish, and my vision became narrower because I was wearing goggles. So, even if I moved a little, it got too hot and I would sweat too much, and it was really hard to deal with something in there. Because it was too hot.*

(Participant D)

#### 3.2.2. Work Loaded Solely on Nurses 

To prevent the spread of COVID-19, hospitals implemented policies to minimize the number of family members and caregivers in contact with patients, which increased the burden of caregiving on participants. Blood collections and portable X-ray imaging that radiological technologists performed also became nurses’ duties. In addition, nurses had to prepare documents for the hospital transfers of patients, and were also responsible for checking, storing, and delivering parcels to patients. Nurses were gradually exhausted as most duties, especially those outside their purview, were delegated to them.

*To be honest, there are not just nurses in the hospital. However, it’s a situation where we have to take on everything that other employees have done. I feel like they’re giving all their work to the nurses. We have to prepare everything that the radiology department had to do on their own before. For the meal distribution for COVID-19 patients, nurses have to do everything that the nutrition team previously did. For blood collection, we have to do all the things that the laboratory medicine department used to do. It’s overwhelming that nurses have to do most of the work.*

(Participant F)

#### 3.2.3. Confusing and Uncertain Working Conditions

Participants’ routine caring for COVID-19 patients has been as uncertain as COVID-19 patients’ conditions. Due to the number of confirmed cases increasing daily and sudden confirmations of the infection in colleagues, situations such as the operation of additional negative pressure wards or temporary closures of wards occurred unexpectedly. Consequently, participants were frequently relocated, and their work schedules and wards were changed, creating confusion. In particular, unclear guidelines and insufficient training made their jobs more difficult.

*It’s tough to get the work schedule on a weekly basis. Actually, I don’t know my work schedule for Tuesday even on Monday, so I don’t know which shift I will work on the next day. Hence, it’s really very stressful.*

(Participant E)

### 3.3. Double Suffering from Patient Care

Participants experienced not only physical difficulties but also mental and social challenges while caring for COVID-19 patients. They endured self-isolation along with their families, and were uncomfortable with causing their family members to experience isolation. In addition, unlike the usual positive public perception of nurses, participants felt a social disconnection from the negativity and stigma surrounding them, which was also hurtful and uncomfortable.

#### 3.3.1. Self-Isolation: Anxiety Becomes a Reality

Participants contracted the virus while caring for patients or had to enter complete self-isolation due to coming in contact with infected colleagues. They endured the anxiety and fear of being infected and suddenly became subjects of self-isolation, leading to concerns about having their personal information exposed, and the social stigma of being confirmed COVID-19 patients. Those who tested negative felt “uncomfortable relief”, even as their colleagues were testing positive during self-isolation.

*When being in self-isolation, as you know, I must contact my child’s school. I had to contact a homeroom teacher of my child. Actually I didn’t really do anything wrong, but I really, really felt bad. Wouldn’t the image appear strange to my child? Because of that thought, every time I thought about that, I thought if I should resign.*

(Participant N)

#### 3.3.2. A Contrasting Perception of Nurses: Heroes of Society versus Subjects of Avoidance

Even with the “Thank you Challenge” campaign spreading among the public, to express gratitude and respect towards health care professionals who responded to COVID-19, nurses did not feel particularly gratified. In a pandemic, the true heroes fighting COVID-19 could only work efficiently in isolation from other people. Close neighbors viewed participants as dangerous sources of pollution or pathogens that threatened their safety. Unlike the warm gaze of the public to see the nurses, participants felt judged by those around them, which made their jobs more uncomfortable.

*Above all, the most challenging thing is the social perspective of “these people are working in an isolation hospital now”. People close to me have this kind of perspective… When one of the nurses is reported on the news or the media as a confirmed patient, we also feel like cringing. Such social perspectives were very hard for us because we’ve become people that the public wants to avoid rather them feeling appreciation for us and thinking of us like we are working hard and trying our best.*

(Participant M)

### 3.4. Support for Caring

Sympathetic colleagues, and supportive and appreciative patients, encouraged participants to care for patients despite their difficulties. In addition, participants felt rewarded and proud of their care when they witnessed patients recovering, which further drove them to fulfill their duties.

#### 3.4.1. Companionship and Sharing Difficulties

Participants endured difficult working routines with the support of colleagues, who best understood their struggles. In experiencing and sharing the same difficulties, participants found comfort with their colleagues. As nurses cannot quit, as that would mean additional pressures for their colleagues, they rely on each other for support.

*To be honest, I think I’m being able to endure hard times thanks to my companionship. It’s hard for us all. And fortunately, all colleagues are friendly, and many colleagues are so considerate of each other. We’re not pushing each other to go in, but we are voluntarily working. Even though COVID-19 is hard for me, this companionship has helped me learn and endure with them until now.*

(Participant I)

#### 3.4.2. Support and Appreciation from Patients and People

While struggling, words of support and appreciation from patients, family, and friends helped participants withstand their difficult situations.

*A patient wrote a very long letter. “Thank you. Thank you so much for taking care of me, and I was moved by the hard work you did. And even in the heat, you never got annoyed”. Well, because the patient wrote a lot of appreciative words like this, I was really grateful. Somehow, apart from the money, I thought it was terrific to work.*

(Participant A)

#### 3.4.3. A Sense of Satisfaction and Self-Esteem

The sense of satisfaction and self-esteem felt while caring for COVID-19 patients became an essential incentive for participants to remain in nursing. When patients hospitalized in severe conditions were able to recover, participants felt rewarded by their occupation, and their self-esteem was increased.

*At first, the patient‘s condition was so bad. So, we thought the patient would actually die, but it turned out that the patient improved so much and was discharged later. We felt like we were being compensated for the hard work. I had pride that we did an excellent job in nursing.*

(Participant D)

### 3.5. Expectations for Post-COVID-19 Life

As COVID-19 keeps persisting in everyday life, expectations for life after COVID-19 are gradually blurring. Participants are unsure if there will ever be a time when they can care for their patients without protective clothing. Much of what participants wanted to accomplish after COVID-19 has been delayed for at least a year, but they have some expectations and are preparing for another future.

#### 3.5.1. Restoring Everyday Life

Even in the current uncertain situation, participants have sincerely performed their nursing duties, while dreaming of restoring daily life. They recognized the importance of everyday social activities, such as eating together, watching movies, capturing bright smiles on camera, and realized that these activities were all they wished to do. Conversely, along with these wishes, there are also concerns about being able to return to the past sense of normalcy.

*Returning to normality is what I want the most, and I think the next step is to think about it together with the management team and the government. I believe our request should be reviewed to combat physical exhaustion, and psychotherapists need to be involved and actively work on recovering. It’s not just that we get rest. Professional intervention is necessary.*

(Participant M)

#### 3.5.2. Preparing for the Future

Participants encountered COVID-19, which occurred several years after the Middle East respiratory syndrome (MERS) epidemic, as another infectious disease that was able to threaten society at any time. In addition, chaotic situations in the hospital were not promptly managed, as the effects of the virus were so severe and fast that the experience of nursing MERS patients became insignificant. The MERS experience was inadequate in training healthcare providers to respond to similar future emergencies. Accordingly, efforts have been made to incorporate the vivid nursing experiences of COVID-19 into protocols against bracing for other diseases in the future.

*That’s why even though I don’t know when the COVID-19 pandemic will end, once it’s over, I think the protocol needs to be more complete. Furthermore, I think we should regularly stockpile a certain amount of items for the future. And, we need to plan a little more neatly how to manage nursing staff systematically.*

(Participant K)

*Since we don’t know when another infectious disease will afflict us, we have to prepare a lot for response training to infectious diseases, facilities and personnel of institutions, and locations for care facilities. To reduce certain mistakes, I think we should prepare well now.*

(Participant M)

## 4. Discussion

This study was conducted to understand the meanings and essence of the experiences of nurses who cared for COVID-19 patients, using a descriptive phenomenological method. As a result of this study, 5 theme clusters and 12 themes were extracted.

The first theme cluster indicated that the nurses struggled under the weight of dealing with infectious diseases. Participants expressed anxiety and fear in the absence of a definitive treatment for COVID-19. This is similar to the results of previous studies that reported that the lack of information and knowledge about unfamiliar diseases leads to ambiguity in nursing services, resulting in nurses feeling fearful and anxious [33]. The anxiety and fear accompanying patient care may be the result of rushing to the battlefield without any preparation [19]. In addition, participants appeared to have persistent fears of unintentional exposure and of transmitting the virus to co-workers [34]. Nurses who performed shift work during COVID-19 had a significantly increased association between COVID-19-related work stressors and anxiety disorder [24]. These physiological and psychological conditions are reported to create high stress and further lead to post-traumatic stress [35]. Hence, nurses caring for COVID-19 patients require continuous evaluation and management to sustain their mental wellbeing. 

In the COVID-19 pandemic, nurses are experiencing ethical anguish in the face of unique situations that they have never experienced before. In particular, watching patients pass away alone, in isolation, without the support and comfort of family members, causes unimaginable shock and anguish. Moral distress between patient dignity and infection control is a similar experience to nurses in other countries, reported in previous studies. Nurses are known to experience contradictory feelings [18] as they experience the pressure of having to coordinate their responsibilities for the prevention of COVID-19 infection, along with other moral responsibilities [16].

Therefore, we need to create an ethically supportive environment [36], not just alleviate the ethical distress experienced by nurses [37]. In addition, it is necessary to find ways to guarantee both infection control and dignified death; for instance, family members can wear protective clothing and safely participate in their relatives’ end-of-life processes. Other measures to ensure a dignified death include minimal post-mortem medical interference, and respect for and adherence to cultural customs [38].

The second theme cluster was participants’ aggravated caring difficulties. Participants in this study were uncomfortable with the heat and sweat caused by wearing sealed PPE. This seems to be a slightly different experience than the Italian nurses who raised some concerns about the lack of PPE, the inadequacy of PPE, and the lack of guidelines for proper use [15]. In Korea, where resources, such as PPE, were relatively abundant since the COVID-19 pandemic declaration, wearing PPE acted as a triple pain burden on the safety of all people rather than the problem of lack of equipment.

It is similar to a previous study, demonstrating that these devices make it difficult to communicate with patients and perform basic tasks [34]. The appropriate wearing of PPE has been reported to protect medical staff from burnout [39]. However, continuous wearing of PPE can cause tissue damage or skin reactions, and prolonged wearing of goggles has been found to increase discomfort and fatigue due to abrasive straps and visual distortion [38]. Therefore, compliance with the PPE-wearing guidelines should be monitored and shift work should be assigned, taking into account the maximum period during which nurses are allowed to wear protective equipment.

It has also been found that medical workload has been excessively delegated to nurses taking care of COVID-19 patients. Policies to minimize social contact with patients have burdened nurses with extra tasks, causing exhaustion [40]. The excessive increase in work burden is in line with the results of qualitative research on the experience of nurses in other countries. A study by Liu et al. [34], in the early days of the COVID-19 pandemic, reported that nurses had done a lot of work. Recent studies also reported that COVID-19 caused a lot of work for nurses [20], and the treatment characterized by many isolated patients increased the work of nurses exponentially [14]. Nurses are constantly aware of new knowledge and skills associated with evolving pandemics and viruses, and receive new training, in preparation for adapting to the situation and providing care for suspected or identified patients [20]. In addition, frequent changes of working locations and wards, changes in work schedules, and confusion over working guidelines, have made nurses’ lives uncertain. 

The final theme of the challenge with difficult care was the confusing and uncertain working conditions, partly related to nursing staffing [14]. However, it was more difficult for the participants in this study to be able to predict their work schedule, rather than the shortage of nursing personnel. This may be due to the difficulty in predicting the hospitalization rates of infected patients and the problems caused by frequent and rapid relocation of nurses, depending on the number of hospitalized patients. In this study, the uncertainty in working conditions is consistent with the report by Liang et al. [20], that there was uncertainty among nurses about being transferred to the areas where the epidemic was most serious. Moreover, the ambiguity surrounding COVID-19 and whether patients have contracted it have been shown to increase nurses’ stress [33]. Even in such situations, thoroughly preparing for and predicting potential emergency situations, based on comprehensive data analysis, knowledge accumulation, and education, can reduce the uncertainty and anxiety surrounding infectious diseases. 

The third theme cluster was double suffering from patient care. Despite continuing to monitor self-health to avoid infecting others, nurses contracted the virus or had to self-isolate due co-workers’ positive diagnoses. Sabetian et al. [41] found that 273 out of a total of 4854 cases contracted the virus while caring for COVID-19 patients, of which 51.3% were nurses. The fear of self-reliance approaching reality is a reflection of the situation at the time, when nurses were not allowed to return home after cohort isolation for two weeks as their colleagues were diagnosed with COVID-19 [19].

Notably, participants felt that they were subjected to dual perceptions, both as national heroes and as contagions. In Korea, the “Thank You Challenge” campaign encouraged expressing gratitude and respect to medical staff. The Korean people were deeply impressed by the situation of nurses and care protection, as they knew that they could not care for patients infected with COVID-19 without the sacrifice and compassionate mission of the nurses [42]. However, nurses have reported preferring forms of recognition and support other than hero worship [37], indicating that the campaign alone was insufficient in improving their morale. Participants also felt that their community members wanted to avoid them and considered them as dangerous contagions, threatening public safety. Previous studies reported that nurses were treated as viruses [19] or suffered from stigma [20], and conversely, were motivated to work harder through public support [19]. However, there are few research reports that nurses experience double suffering from patient care due to the coexistence of such contrasting perceptions. These experiences corroborate previous findings that disease uncertainty and social anxiety have caused nurses to be perceived as carriers and spreaders of the virus [33].

The fourth theme cluster was supporting caring. Participants endured their situations because quitting would have overburdened their colleagues. While participants found it awkward to work with nurses from different wards at the beginning of the COVID-19 pandemic, their relationships improved and became encouraging and supportive [19]. It is worth noting that, even in situations of extreme stress and emotional exhaustion, support from colleagues and teams can positively impact recovery [43]. In addition, this study found that support and appreciation from patients and families encouraged participants to endure their difficult situations [19,35]. In previous studies, negative emotions, such as fatigue, helplessness, and fear of infections, prevailed in the early stages of COVID-19, but coping strategies were created with adaptation, support from others, and expressions of positive emotions [44]. International researchers reported that nurses dealt with and attempted to overcome their challenges and feelings and emotional responses by coping during the pandemic. Nurses in the United States [17] and India [45] used teamwork and peer support, and used personal coping strategies, such as relationship development, play, exercise, meditation, and distractions.

In the face of unknown diseases and unpredictable dangers, participants took responsibility and devoted themselves to their mission. Despite nurses and healthcare staff demonstrating professional devotion [33,34], a social atmosphere that demands sacrifice should be avoided to decrease their experiences of stress and fatigue.

The last theme cluster encompassed expectations for post-COVID-19 life. The participants had been doing their best to care for patients, while dreaming of returning to their regular lives, despite working in uncertain conditions. To instill a sense of normalcy in their lives, it is imperative to provide physical and mental health support to exhausted nurses. Even after the impact of COVID-19 has diminished, it is necessary to fully recognize the inherent stress and emotional burden experienced by nurses and support recovery with routine procedures and systems [44]. This aspect of the pandemic has been reported by Italian nurses to have obvious psychological trauma, which is quite similar to that reported in China [46,47]. As COVID-19 cases begin to decline, research into resilience, particularly post-traumatic stress syndrome in nursing staff, will be needed [48]. Although new epidemic outbreaks cannot be prevented, risk awareness can direct attention to emerging epidemics and promote capacity development toward disease management and control [19,49]. As seen from this study, experience alone did not prepare nursing staff to deal with novel disease outbreaks. Hence, specific protocols and standard operating procedures, targeting different disease risk scenarios, should be established to support nursing work, with ample resources.

### Limitations of This Research

In this study, we applied a phenomenological approach to understanding nurses’ experiences of COVID-19 patient caring, and the participants were the nurses who involuntarily cared for COVID-19 patients. Accordingly, there is a limitation in that the nursing experience of the nurses who voluntarily participated in COVID-19 patient nursing could not be presented. We conducted online or face-to-face interviews, depending on the participants’ preferences, but the online interview had limitations, in that it did not fully grasp the vivid experiences contained in the non-verbal expressions of the participants and did not describe their experiences in more depth. Participants were in a vulnerable situation; not only were they at risk of infection, but were also responsible for covering the duty of their colleagues with confirmed COVID-19, and the work of other health care assistants because they were wearing PPE. Despite these limitations, it is significant that this study gained a deeper understanding of nurses’ experiences of caring for COVID-19 patients and came a little closer to the essence of nursing.

## 5. Conclusions

This study is significant as it explored and organized nurses’ experiences of caring for COVID-19 patients, using a descriptive phenomenological research method. The findings of this study are useful primary data for developing appropriate measures for health professionals’ wellbeing during outbreaks of infectious diseases.

A limitation of this study is that, because data were collected before the participants were vaccinated against COVID-19, negative emotional aspects, such as anxiety and fear about caring for patients, were drawn as the main results. In the future, it is necessary to balance this perspective by incorporating experiences of healthcare providers who have been vaccinated against COVID-19. In addition, as nurses in this study struggled with mental as well as physical difficulties, it is suggested that future studies develop and apply mental health recovery programs for them.

## Figures and Tables

**Table 1:** General Characteristics of Participants (*N* = 14).

Variables		N
Sex	Male	2
	Female	12
Age (years)	<30	5
	30–39	3
	40–49	6
Education	College	11
	Graduate School	3
Number of patients per nurse	3	1
	4	6
	5	4
	6	2
	7	-
	8	-
	9	1
Period of working in isolation ward, (months)	<3	1
	3–<6	4
	6–<9	5
	9–<12	3
	12≤	1
Change of place of residence during working in the isolation ward, yes		4
Infection control education on COVID-19, yes		12

Note. COVID-19 = coronavirus disease-2019.

**Table 2:** Theme Clusters and Themes.

Theme Clusters	Themes
1. Nurses struggling under the weight of dealing with infectious disease	Anxiety and fear accompanying patient care Dignity ignored due to the fear of infectious diseases
2. Challenges added to difficult caring	The burden of triple distress for everyone’s safety; Wearing PPE Work loaded solely on nurses Confusing and uncertain working conditions
3. Double suffering from patient care	Self-isolation: anxiety becomes a reality A contrasting perception of nurses: heroes of society versus subjects of avoidance
4. Support for caring	Companionship and sharing difficulties Support and appreciation from patients and people A sense of satisfaction and self-esteem
5. Expectations for post-COVID-19 life	Restoring everyday life Preparing for the future

## Data Availability

The data presented in this study are available on request from the corresponding author.
